# The prognostic utility of soluble fms-like tyrosine kinase-1 (sFlt-1) and placental growth factor (PIGF) biomarkers for predicting preeclampsia: a secondary analysis of data from the INSPIRE trial

**DOI:** 10.1186/s12884-022-04817-6

**Published:** 2022-06-27

**Authors:** Meron M. Kifle, Prabin Dahal, Manu Vatish, Ana Sofia Cerdeira, Eric O. Ohuma

**Affiliations:** 1 Centre for Tropical Medicine and Global Health, Nuffield Department of Clinical Medicine, University of Oxford, Oxford, UK; 2 Nuffield Department of Women’s and Reproductive Health, University of Oxford, Oxford, UK; 3 Maternal, Adolescent, Reproductive and Child Health Centre, Department of Infectious Disease Epidemiology, London School of Hygiene and Tropical Medicine, London, UK

**Keywords:** Prognostic model, Preeclampsia, sFlt-1, PIGF, INSPIRE trial

## Abstract

**Objective:**

To compare the prognostic performance of biomarkers soluble fms-like tyrosine kinase-1 (sFlt-1), Placental Growth Factor (PIGF), and sFlt-1/PIGF ratio as continuous values or as a binary cut-off of 38 for predicting preeclampsia (PE) within 7 days.

**Design:**

Secondary analysis of a randomised clinical trial.

**Setting:**

Oxford University Hospitals, Oxford, United Kingdom (UK).

**Population:**

Pregnant women between 24^+0^ to 37^+0^ weeks of gestation with a clinical suspicion of preeclampsia.

**Main outcome:**

Onset of preeclampsia within 7 days of the initial biomarker test.

**Methods:**

Logistic regression model for onset of preeclampsia using: (i) sFlt-1 (ii) PIGF, (iii) sFlt-1/PIGF ratio (continuous), and (iv) sFlt-1/PIGF ratio as a cut-off above or below 38.

**Results:**

Of the total 370 women, 42 (11.3%) developed PE within 7 days of screening. Models with sFlt-1 and sFlt-1/PIGF ratio (continuous) had greater overall performance than models with PIGF or with sFlt-1/PIGF ratio as a cut-off at 38 (*R*^2^: sFlt-1 = 55%, PIGF = 38%, sFlt-1/PIGF ratio = 57%, sFlt-1/PIGF ratio as cut-off at 38 model = 46%). The discrimination performance was the highest in sFlt-1 and sFlt-1/PIGF ratio (continuous) (c-statistic, sFlt-1 = 0.94, sFlt-1/PIGF ratio (continuous) = 0.94) models compared to PIGF or sFlt-1/PIGF cut-off models (c-statistic, PIGF = 0.87, sFlt-1/PIGF cut-off = 0.89).

**Conclusion:**

Models using continuous values of sFlt-1 only or sFlt-1/PIGF ratio had better predictive performance compared to a PIGF only or the model with sFlt-1/PIGF ratio as a cut-off at 38. Further studies based on a larger sample size are warranted to substantiate this finding.

## Introduction

Preeclampsia (PE), defined as a new onset of hypertension and proteinuria usually occurring after 20 weeks of gestation up to and after delivery [1], confers a significant burden on maternal and fetal health outcomes. Globally, 2 to 5% of pregnancies suffer from PE leading to 76,000 maternal and more than 500,000 fetal deaths annually [2]. In the United Kingdom (UK), the incidence of mild PE remains very low (6 per 1,000 pregnancies) while severe PE occurs in around 1 to 2% of pregnancies [3, 4].

To date, supportive management remains the current standard of care for treating PE as there are no therapeutic interventions other than delivery. The pathophysiological mechanisms underlying PE highlight an angiogenic imbalance, reflected by elevated placenta-derived soluble fms-like tyrosine kinase-1 (sFlt-1) and decreased placental growth factor (PIGF) levels in the maternal circulation [5–7]. These biomarkers have been successfully used for diagnosis, prediction of the disease [6–9] and clinical decision making with a combination of other clinical parameters such as high blood pressure and proteinuria [3]. Nevertheless, latest evidence indicates that the ratio of the biomarkers is elevated but its predictive value in women with suspected preeclampsia is unclear [5].

Prognostic models based solely on the classical clinical risk factors (high blood pressure and proteinuria) have been reported to have poorer predictive ability compared to the models developed using sFlt-1 and PIGF biomarkers [10, 11]. However, most of the prognostic models built using sFlt-1, PIGF, or their ratio are based on dichotomising the continuous measurements of these markers using threshold values [4, 5, 12–15]. Moreover, only few studies have modelled or compared sFlt-1, PIGF, or their ratio on a continuous scale [16–18].

Binary thresholds, whilst simpler for clinical application [19], may lead to a loss in statistical power and lead to models with poor predictive performance [20]. In addition, an explicit comparison of the predictive performance of continuous values of sFlt-1, PIGF biomarkers or their ratio against cut-off-based models for predicting PE currently remains uncertain. This study aimed to bridge these gaps by applying a probabilistic approach that uses the mathematical ratio of sFlt-1 and PIGF biomarkers as continuous as opposed to a simplistic discrete (rule-in/rule-out) approach based on a defined threshold obtained from the mathematical ratio of sFlt-1 and PIGF. Specifically, we aimed to compare the prognostic utility of models using the continuous values of sFlt-1, PIGF, or sFlt-1/PIGF ratio for predicting PE within 7 days of screening among those with suspected PE compared to the recommended cut-off-based value of the ratio of sFlt-1/PIGF of 38.

## Methodology

### Data source: the INSPIRE trial

Data from the prospective, parallel group, randomized interventional study evaluating the short-term prediction of preeclampsia/eclampsia (INSPIRE) trial was used for the purpose of this research [21]. The INSPIRE trial was conducted in the UK that aimed to evaluate the use of sFlt-1/PIGF ratio in women presenting with suspected preeclampsia and its effect on PE-related hospitalisation within 24 h of the test, within 7 days, or by delivery as the primary outcome. The study was conducted from June 2015 to April 2017 at the John Radcliffe Hospital, Oxford, UK—a tertiary referral centre with a preeclampsia prevalence of 2.9%.

The study enrolled 370 pregnant women 186 reveal trial arm (standard clinical management plus revealing biomarker results) versus 184 non-reveal trial arm (standard clinical management) aged 18 years or above, with singleton pregnancies between 24^+0^ and 37^+0^ weeks of gestation with a clinical suspicion of preeclampsia. Women with pre-existing diagnosed preeclampsia/eclampsia were excluded from the trial. Suspicion of preeclampsia was defined by a new onset elevated blood pressure or worsening of pre-existing hypertension or new-onset proteinuria or worsening of pre-existing proteinuria or new-onset headache, visual disturbance, oedema or right upper quadrant pain, or any other clinical suspicion of preeclampsia [21].

Overall, there were 85 women with PE until delivery and 42 had PE within 7 days of screening. The study found that there was no difference in preeclampsia-related admissions within 24 h of the test between trial arms (sixty patients were admitted in the intervention group (reveal trial arm) and 48 in the comparator group (non-reveal trial arm).

### Primary outcome and candidate predictors

The primary outcome in this study was the onset of PE within 7 days of the initial biomarker test as defined in the INSPIRE trial [21]. After informed consent, study participants had standard clinical assessment and additional blood sample for biomarker measurement were collected and centrifuged within 1 h of collection. The sFlt-1 and PIGF values were then measured using the fully automated methods (Elecsys® sFlt-1/PIGF) using the Roche e411 analyzer (Roche Diagnostics Limited, Burgess Hill, United Kingdom) [22].

A logistic regression model for predicting the onset of PE within 7 days of screening was done using biomarkers sFlt-1 (continuous values), PIGF (continuous values), sFlt-1/PIGF ratio (continuous values), or sFlt-1/PIGF ratio as a binary cut-off of 38.

### Sample size assessment for the development of a prognostic model

The adequacy of sample size was assessed using the ***pmsampsize*** library in R program as recommended by Riley et al. [23]. The minimum events per parameter required for reliably developing a new model that achieved the desired shrinkage factor, *R*^2^, and margin of difference was ~ 10 events per variable. Therefore, with 42 outcome events, ~ 3–4 variables could be used for model development.

### Multivariable model building

Four multivariable logistic regression models were constructed for the three biomarkers (sFlt-1, PIGF, sFlt-1/PIGF ratio as continuous, and sFlt-1/PIGF ratio cut off at 38). All four models were adjusted for trial arm [24]. Natural log transformation was carried out for continuous values of sFlt-1, PIGF and sFlt-1/PIGF ratio. The sFlt-1/PIGF cut-off used was 38 since it is commonly used by many studies including the INSPIRE trial [22]. The multivariable logistic regression model assumed the log-odds of PE were linearly associated with the biomarkers. This assumption was formally tested by making comparisons with fractional polynomial regression models. Models were compared using the likelihood ratio test and Bayesian Information Criterion.

### Model performance and internal validation

The predictive performances of the four developed models were assessed using calibration and discrimination. Model calibration was assessed by calibration-in-the-large (CITL) and calibration slope whereas model discrimination was assessed by Harrell’s concordance statistic (c-statistic) [25]. The best performing models were tested formally by comparing the respective area under the curve (AUC) using the Delong test [23, 25]. Overall model fit was assessed using Pseudo (Nagelkerke’s) *R*^2^ and Bayesian Information Criterion (BIC) [25]. The apparent performance measures (model fit performance in the development data) were adjusted for optimism using bootstrapping by drawing 1,000 resamples from the original dataset and calculating adjusted measures of concordance statistic (calculated from the Somers’ D rank correlation value) [23], calibration slope and calibration-in-the-large. Adjusted coefficients were corrected for optimism using the uniform shrinkage factor i.e., the calibration slope obtained from bootstrapping. The intercept of the optimism-adjusted model was also re-estimated to maintain the overall calibration. The transparent reporting of a multivariable prediction model for individual prognosis or diagnosis (TRIPOD) guideline was used for model development and reporting [26, 27].

## Results

### Baseline characteristics of the study population

There were 370 study participants of whom 42 (11.3%) were diagnosed with preeclampsia within 7 days of taking the screening test. Baseline characteristics for women with and without PE were similar except for gestational age at recruitment and parity (Table 1). The distributions for PIGF, sFlt-1, and the ratio sFlt-1/PIGF were skewed and therefore were transformed using natural logarithms.

**Table 1:** Baseline characteristics of study population (*n* = 370)

Characteristics	Preeclampsia within 7 days (***n*** = 42)	No preeclampsia within 7 days (***n*** = 328)
**Maternal age at recruitment** (in years), Mean (SD)	31.2 (5.8)	31.2 (6.1)
**Gestational age** (in weeks), Median (IQR)	35.3 (33.4—36.1)	34.1 (31.1—35.9)
**BMI** (in kg/m^2^), Median (IQR)	27.3 (24.5—31.7)	27.3 (23.9—32)
**Parity**		
Nulliparous	35 (83.3%)	145 (44.2%)
Multiparous	7 (16.7%)	183(55.8%)
**Smoking status**		
Current smoker	2 (4.8%)	31 (9.4%)
Never smoker	24 (57.1%)	201 (61.3%)
Previous smoker	16 (38.1%)	96 (29.3%)
**Ethnicity**		
White British	37 (88.1%)	295 (89.9%)
Other	5 (11.9%)	33 (10.1%)
^a^ **sFlt-1**, Median (IQR)	4.03 (3.88—4.15)	3.38 (3.18 – 3.61)
^a^ **PIGF**, Median (IQR)	1.87 (1.72 – 2.07)	2.40 (2.17 -2.73)
^a^ **sFlt-1/PIGF ratio**, Median (IQR)	2.11 (1.82 – 2.35	0.89 (0.56 – 1.44)
**sFlt-1/PIGF cut off**		
Ratio ≤ 38	1 (2.4%)	256 (78.1%)
Ratio > 38	41 (97.6%)	72 (21.9%)

*SD* Standard deviation, *PIGF* Placental growth factor, *sFlt-1* Soluble fms-like tyrosine kinase-1, *IQR* Interquartile range, *BMI* Body mass index, *BP* Blood pressure

^a^sFlt-1, PIGF and the sFlt-1/PIGF ratio reported on natural log scale

The median ln(PIGF) was higher among non-preeclamptic women (median: 2.40 pg/mL; interquartile range (IQR): 2.17 pg/mL–2.73 pg/mL) compared to preeclamptic women (median: 1.87 pg/mL; IQR: 1.72 pg/mL – 2.07 pg/mL). The median ln(sFlt-1) for women without PE was 3.38 pg/mL (IQR: 3.18 pg/mL – 3.61 pg/mL) compared to 4.03 pg/mL (IQR: 3.38 pg/mL – 4.15 pg/mL) for preeclamptic women. Similarly, for ln(sFlt-1/PIGF) ratio, the median was 0.89 (IQR: 0.56 – 1.44) among non-preeclamptic women compared to 2.11 (IQR: 1.82 – 2.35) for preeclamptic women. The sFlt-1/PIGF ratio values ≤ 38 were present in 78% of women without PE while 21.9% of women without PE had sFlt-1/PIGF cut-off values > 38 compared to 97.6% in women with PE (Table 1). The sFlt-1/PIGF ratio values > 85 was present in 24 (7.3%) women without PE compared to 29 (69%) women with PE. Considering sFlt-1/PIGF ratio values between 38 to 85, 12 (28.6%) women had PE compared to 48 (14.6%) women without PE (data not shown).

### Model development and performance

The fitted models are presented in Table 2 and the performance measures are presented in Table 3. The sFlt-1 (*R*^2^ = 55%, BIC = 144) and sFlt-1/PIGF ratio models (*R*^2^ = 57%, BIC = 139) showed higher overall model fit than PIGF model (*R*^2^ = 38%, BIC = 184) or sFlt-1/PIGF ratio using a binary cut-off of 38 model (*R*^2^ = 46%, BIC = 166).

**Table 2:** Details of the four multivariable logistic regression models fitted

Models	Model form
Model 1	$PE\sim\log\left(sFlt-1\right)+randomised\ arm$
Model 2	$PE \sim \mathrm{log}(PIGF)+randomised\ arm$
Model 3	$PE \sim log\left(\frac{sFlt-1}{PIGF}\right)+randomised\ arm$
Model 4	$PE \sim \left(\frac{\mathrm{sFlt}-1}{\mathrm{PIGF}}\right)>38 +randomised\ arm$

*PE* Pre-eclampsia (1 = Yes, 0 = No), *sFlt-1* Soluble fms-like tyrosine kinase – 1, *PIGF* Placental growth factor, *log* indicates natural logarithmic transformation

**Table 3:** Performance measures of biomarkers in identifying preeclampsia

Model	Regression model output		Model performance corrected for optimism					
	Coefficient	Intercept	Calibration slope	CITL	C-index	*R*^2^	BIC	AUC
Model 1: sFlt-1 only^a^	7.37	-29.92	0.98	0	0.94	0.55	144	0.94
Model 2: PIGF only^a^	-4.77	8.08	0.99	0	0.87	0.38	184	0.89
Model 3: sFlt-1/PIGF ratio^a^	-9.47	4.19	0.99	0	0.94	0.57	139	0.94
Model 4: sFlt-1/PIGF cut-off at 38^b^	4.37	-5.81	0.84	0	0.88	0.46	165	0.89

*sFlt-1* Soluble fms-like tyrosine kinase – 1, *PIGF* Placental growth factor, *CITL* Calibration-in-the-large, *BIC* Bayesian information criteria (the lowest BIC values indicating better model performance), *C-index* Concordance index, *AUC* Area under the curve

^a^Predictor variables are continuous values included in the model equation of biomarker/s as every unit increases in log-scale. All model results are optimism corrected and adjusted for the trial arm

^b^No log transformation applied for the cut off predictor variable

**Fig. 1:**
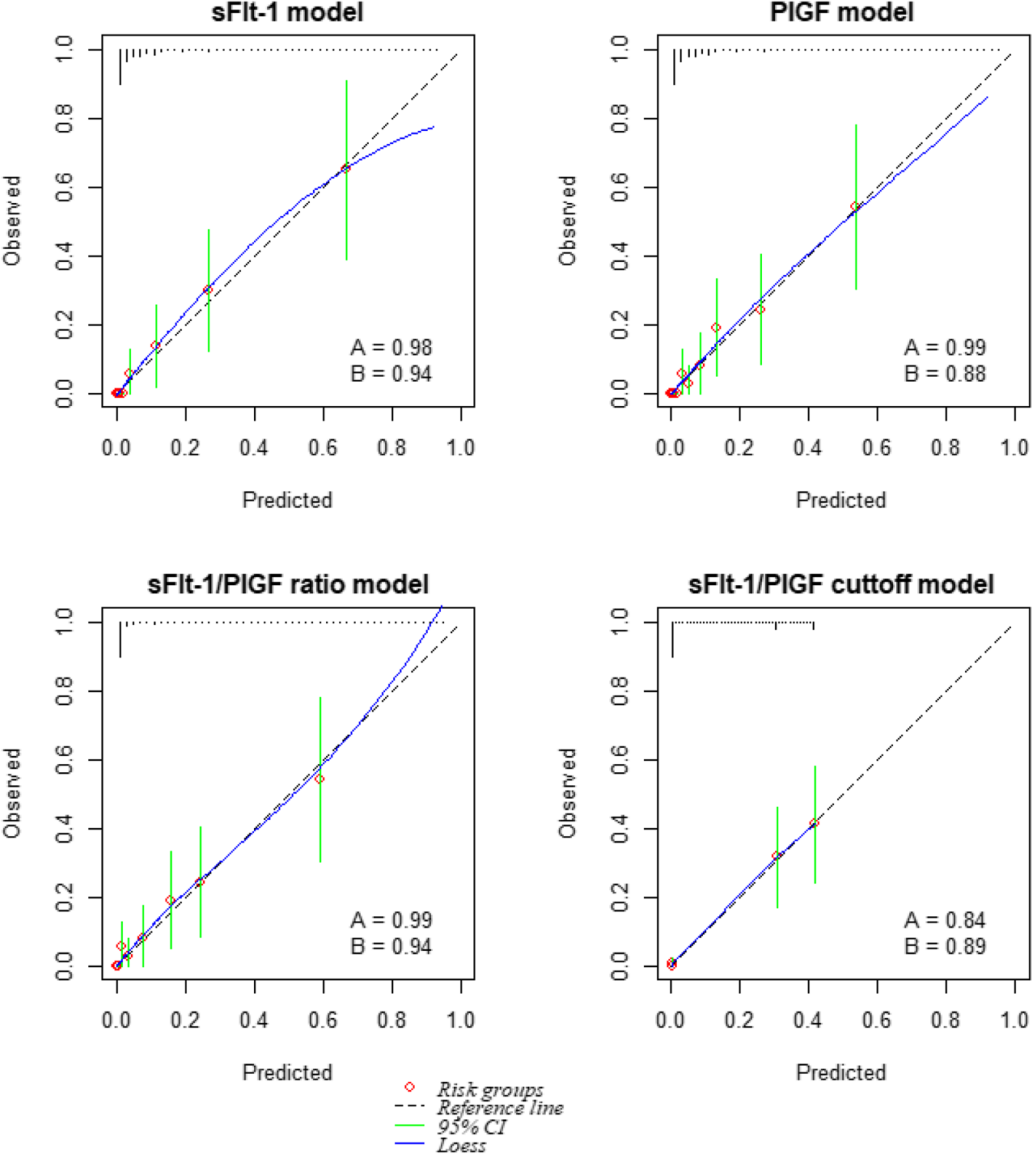
Assessment of model calibration for sFlt-1, PIGF, sFlt-1/PIGF ratio (continuous) and sFlt-1/PIGF cut-off-based models. sFlt-1, PIGF and sFlt-1/PIGF ratio (continuous) values are in log scales. Estimated probabilities are plotted in ten risk groups. The plot for sFlt-1/PIGF cut-off-based model is based on the log odds of predictions of the 0/1 outcome. A = calibration slope, B = c-statistic, CI - confidence interval, Loess - Locally weighted polynomial regression

The mean predictions (CITL) for PE were close to zero for all models (Table 3). The sFlt-1, PIGF and sFlt-1/PIGF ratio (continuous) models all had calibration slope close to 1 (slope >  = 0.98) except for sFlt-1/PIGF cut-off of 38 model (slope = 0.84) (Fig. 1 and Table 3). Model discrimination was ≥ 0.87 across all models (c-statistic for: sFlt-1 = 0.94, PIGF = 0.88, sFlt-1/PIGF ratio = 0.94, sFlt-1/PIGF ratio using a binary cut-off of 38 model = 0.89) (Table 3).

**Fig. 2:**
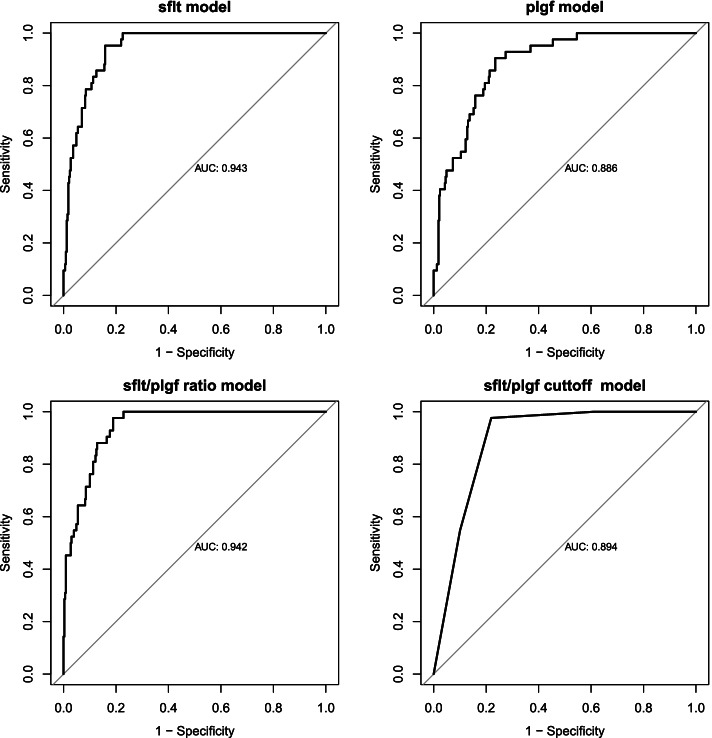
Assessment of area under the curve for sFlt-1, PIGF, sFlt-1/PIGF ratio (continuous) and sFlt-1/PIGF cut-off-based models

There was a statistically significant difference in the AUC for sFlt-1 model (AUC = 0.94) compared to PIGF model (AUC = 0.89), *p*-value = 0.013. The sFlt-1/PIGF ratio model was better than PIGF (AUC = 0.94 vs 0.89), *p*-value = 0.001 and sFlt-1/PIGF cut-off model (AUC: 0.94 vs 0.89), *p*-value = 0.001. The sFlt-1 model and sFlt-1/PIGF ratio models had the best and similar AUCs of 0.94 (Fig. 2).

## Discussion

### Main findings

This study compared the discriminative ability of sFlt-1, PIGF, sFlt-1/PIGF ratio as continuous, and sFlt-1/PIGF ratio using a binary cut-off of 38 for identifying preeclampsia within 7 days of screening. The continuous values of biomarkers sFlt-1 alone and sFlt-1/PIGF ratio had comparable predictive performance with similar discrimination ability to identify PE cases. These performed better than PIGF alone or sFlt-1/PIGF cut-off model. The commonly used sFlt-1/PIGF ratio using a binary cut-off of 38 had poorer predictive performance relative to other models considered. This finding was in line with the fact that biomarkers perform better as continuous variables than as dichotomous cut-offs as it is more biologically plausible because the disease profile is a continuum [17], and in particular in preeclampsia, where the biomarkers are related to its pathogenesis [28]. This finding highlights the need for further studies to evaluate approaches for handling biomarkers (continuous versus cut off) for identifying PE.

### Interpretation

Studies that utilise continuous values of biomarkers for the prediction of preeclampsia are limited as many biomarker-based prediction studies often employ cut off values [10] or predict maternal outcomes after the diagnosis of preeclampsia [29]. A systematic review and meta-analysis of the sFlt-1/PIGF ratio cut off for prediction of PE pointed out that the ratio-based model has a good predictive potential, but conclusive evidence is lacking because of the differences in the choice of cut-offs used, timing and frequency of testing, and due to heterogeneity in the target population [10]. Saleh et al. compared the continuous versus cut-off-based biomarker-based prediction of pregnancy complications including preeclampsia [17] and found that continuous value of sFlt-1/PIGF ratio had high discrimination performance with sFlt-1 and PIGF cut off values having lower predictive ability than the continuous biomarker values. Perry et al. [30] also showed that continuous values of sFlt-1/PIGF ratio performed better than cut off based predictions with an additive value from baseline clinical predictors. Our findings were consistent with the findings of Saleh et al. and Perry et al. However, these previous studies had a target population of pregnancies already complicated with preeclampsia, gestational hypertension, or chronic hypertension; making direct comparisons difficult because the study participants included in the INSPIRE trial were only women suspected with preeclampsia [21].

The models for sFlt-1 or sFlt-1/PIGF ratio as continuous had similar performance in terms of calibration and discrimination, indicating no significant advantage in using ratio values of sFlt-1 and PIGF biomarkers (as continuous) to using sFlt-1 only as a biomarker. This finding concurs with the results reported in the preeclampsia prediction literature [10, 16]. For instance, Anderson et al. found that for early-onset and severe preeclampsia cases, the PIGF model performed similarly to the sFlt-1/PIGF cut-off model indicating no meaningful advantage over using cut off based prediction. This is consistent with the results of a systematic review [8] that reported PIGF had moderate-to-high evidence for identifying women at the highest risk of preterm delivery or neonatal outcomes but no clinically useful performance for the prediction of adverse maternal outcomes. This might be physiologically plausible as PIGF levels are associated with other non-placental factors and intrauterine growth restriction; in contrast to sFlt-1 levels which have a strong correlation with the placenta [31]. Furthermore, free PIGF levels seem to be decreased in preeclampsia mostly because of sFlt-1 binding [32]. Nevertheless, Ukah et al. did recognise that it is not clear if PIGF performs better alone or in combination, and more studies are needed to assess this [8]. In real-world clinical practice, the use of PIGF is reported to have a predictive potential for delivery of small for gestational age infants [33] and lower time for preeclampsia diagnosis by clinicians [9].

### Strengths and limitations

Our study has several strengths and limitations. The predictive ability of the biomarkers was assessed as continuous variables as recommended in prognostic model development [19]. Prior to starting modelling, the recommended test of sample size adequacy was performed and only parameters with the minimum events were included, indicating the relevance of the parameters used for model building. Adjustment of model performance parameters by bootstrapping also increased the likelihood of having realistic model performance measures.

However, the INSPIRE trial was a single centre study and the institution-specific level practices and overall context might affect the generalisability of the study findings. Finally, the small number of events in our study limited the scope of constructing a multivariable model with other potentially important parameters such as age, parity, and BMI.

## Conclusion

Using continuous values of either sFlt-1/PIGF ratio or sFlt-1 alone had better predictive performance over models based only on continuous values of PIGF only and sFlt-1/PIGF cut-off at 38. There was no obvious incremental value in using sFlt-1 only or continuous values of sFlt-1/PIGF ratio values as they both had comparable predictive performance. More studies with larger numbers of patients and sufficient outcome variables are warranted to evaluate whether the ratio cut-off of 38 or an alternative that is based on a continuous value of a single biomarker or ratio can perform better in predicting PE.

## Data Availability

The datasets used and analysed during the current study are not publicly available due to data privacy for identifiable patient information and ethical concerns but are available from the corresponding author on reasonable request.

## Abbreviations

CITL: Calibration-in-the large
c-statistic: Concordance statistic
INSPIRE: Interventional Study Evaluating the Short-Term Prediction of Preeclampsia/Eclampsia
IQR: Interquartile range
PE: Preeclampsia
PIGF: Placental growth factor
RMSE: Root mean square error
SD: Standard deviation
sFlt-1: Soluble fms-like tyrosine kinase-1
TRIPOD: Transparent reporting of a multivariable prediction model for individual prognosis or diagnosis
